# Psychoeducational Characteristics of Children with Hypohidrotic Ectodermal Dysplasia

**DOI:** 10.1100/2012/532371

**Published:** 2012-03-12

**Authors:** Rolanda A. Maxim, Samuel H. Zinner, Hisako Matsuo, Theresa M. Prosser, Mary Fete, Terry L. Leet, Timothy J. Fete

**Affiliations:** ^1^Division of Developmental Pediatrics, Department of Pediatrics, Saint Louis University, St. Louis, MO 63014, USA; ^2^Division of Developmental Medicine, Department of Pediatrics, University of Washington, Seattle, WA 98195-5852, USA; ^3^Department of Sociology and Criminal Justice, Saint Louis University, St. Louis, MO 63108, USA; ^4^School of Education, Texas Christian University, Forth Worth, TX 76129-0002, USA; ^5^National Foundation for Ectodermal Dysplasias, Mascoutah, IL 62258, USA; ^6^Department of Pediatrics, Saint Louis University, St. Louis, MO 63108, USA; ^7^Department of Community Health, Saint Louis University, St. Louis, MO 63108, USA; ^8^Department of Child Health, University of Missouri, Columbia, MO 65212, USA

## Abstract

*Objective*. Hypohidrotic ectodermal dysplasia (HED) is an X-linked hereditary disorder characterized by hypohidrosis, hypotrichosis, and anomalous dentition. Estimates of up to 50% of affected children having intellectual disability are controversial. *Method*. In a cross-sectional study, 45 youth with HED (77% males, mean age 9.75 years) and 59 matched unaffected controls (70% males, mean age 9.79 years) were administered the Kaufman Brief Intelligence Test and the Kaufman Test of Educational Achievement, and their parents completed standardized neurodevelopmental and behavioral measures, educational, and health-related information regarding their child, as well as standardized and nonstandardized data regarding socioeconomic information for their family. *Results*. There were no statistically significant differences between the two groups in intelligence quotient composite and educational achievement scores, suggesting absence of learning disability in either group. No gender differences within or between groups were found on any performance measures. Among affected youth, parental education level correlated positively with (1) cognitive vocabulary scores and cognitive composite scores; (2) educational achievement for mathematics, reading, and composite scores. *Conclusion*. Youth affected with HED and unaffected matched peers have similar profiles on standardized measures of cognition, educational achievement, and adaptive functioning although children with HED may be at increased risk for ADHD.

## 1. Introduction

Hypohidrotic ectodermal dysplasia (HED) is the most common of a group of related heritable disorders of the embryonic ectoderm, characterized by congenital absence or abnormal function of two or more ectodermal structures and their accessory appendages. Features may include a lack of sweat glands (hypohidrosis), alopecia (hypotrichosis), anomalous dentition, hypodontia [1], abnormal tooth structure, nail dystrophy (onychodysplasia), defective palms and soles (palmoplantar hyperkeratosis), and other features [2–5]. Genetic inheritance is X-linked recessive among the majority of affected people, with only a small minority of cases arising from autosomal recessive or autosomal dominant inheritance. Genetic testing is available, and, in the X-linked form, DNA sequence analysis of the coding region detects mutations in about 95% of affected males and a lower percentage of carrier females [6]. Diagnosis is usually based on recognizable and common clinical features. 

While defects in skin and other tissues of ectodermal origin are well-established consequences of HED, it is not clear whether youth with HED are at increased risk for cognitive and related neurological and/or adaptive impairment. Some studies assessing cognition in people with HED have suggested substantial risk (10–50%) for delayed mental development [7–10]. A number of factors may contribute to these impressions, including bias toward people with facial or dental dysmorphic features [11], unexamined impressions of academic performance or intelligence [12, 13], and increased risk for hyperthermic brain damage [14]. However, well-designed cognitive and academic psychometric assessments of children with HED have not been published, despite awareness for decades of this research gap and concerns regarding the potential for unchallenged misleading assumptions [15].

There are roughly 200 identified distinct non-HED ectodermal dysplasias and associated disorders, many with overlapping clinical phenotypic features as HED [16] and some of which have been associated with intellectual disability [1, 17–20]. In addition, the embryonic ectodermal germ layer gives rise to brain and skin organ systems, and many neurocutaneous (brain-skin) disorders are highly associated with cognitive and behavioral impairment, such as neurofibromatosis and tuberous sclerosis. Further, there is potential for brain insult resulting from high fever [7, 8], a consequence of hypohidrosis. Chronic and recurrent medical complications may necessitate frequent school absence [21]. These and other variables place youth with HED at increased risk for impairment to psychosocial, cognitive, academic, and adaptive function [21].

In contrast, case reports indicate above average cognitive skills among tested adults with HED, and some adults with HED have earned college degrees in engineering and in economics [8]. Typical adaptive functioning in children has also been described [21]. However, these and related reports are sparse and limited by small sample size and other methodological weaknesses. Data relating age, socioeconomic and gender variables to psychoeducational performance in children with HED have not been reported. In addition, population risk for attention deficit hyperactivity disorder (ADHD) and/or learning disability in children with HED has not been assessed.

Our objectives were to assess youth with HED for (1) characteristics of cognitive, educational achievement, and adaptive skills; and (2) association with ADHD. Our first hypothesis was that children with HED would have lower cognitive and educational achievement performance on standardized instruments when compared with unaffected controls. Our second hypothesis was that, according to parental report, youth with HED would have a higher prevalence of signs of ADHD and of difficulty with adaptive functioning when compared with unaffected controls.

## 2. Methods

### 2.1. Study Design

Our study used a cross-sectional design to assess psychoeducational performance and related characteristics of children with HED. For our control group, we selected children and their younger siblings from two parochial schools in St. Louis, Missouri. Assessments included administration of standardized instruments to all participating youth, as well as parent-completed indirect measures. The Saint Louis University Institutional Review Board for Human Subject Research approved the study.

### 2.2. Recruitment

We recruited and tested separately youth with HED and control subjects frequency matched for age, gender, and socioeconomic status (SES). For recruitment of children diagnosed with HED, prior to the annual conferences of the National Foundation of Ectodermal Dysplasias (NFED) held in July 2002 and July 2003 in Salt Lake City, Utah, and Iselin, NJ, respectively, registered parents were mailed a flyer that provided information regarding the study with an invitation to enroll. All youth with HED attending the conference aged 4–19 years and without profound hearing and/or vision deficits were eligible for participation. The HED diagnosis was based on parental report. Children serving as control subjects were recruited at two parochial schools for children ages 5 to 14 years in Saint Louis, Missouri, during August–December 2004. Control children were selected to match for age and SES distribution of participating children represented in the HED group. Before the evaluation of the children, a package containing an invitation letter, a consent form, a General Information Questionnaire (GIQ), a Conners' Parent Rating Scale-Revised: Short Version (CPRS-R:S), and an Adaptive Behavior Assessment Scale (ABAS) was given to the parents of children attending the NFED conference and was also mailed to parents of children participating in the control group. Before the enrollment, all participating children and their parents signed consent/assent forms.

### 2.3. Instruments

All children were assessed for verbal, nonverbal, and full-scale intelligence; educational achievement in reading, mathematics, and spelling; adaptive measures; attention, hyperactivity, and oppositional behavior; general medical and educational history; family-based socioeconomic and caregiver occupational and educational status using the following instruments.

*Adaptive Behavior Assessment System: Parent Form* (ABAS), designed to assess youth ages 5–21 years, is a standardized parent-response instrument used for comprehensive assessment in ten areas of adaptive skills specified by the American Association on Intellectual and Developmental Disabilities (AAIDD) and the Diagnostic and Statistical Manual of Mental Disorders, 4th edition, Text Revision (DSM-IV-TR) [22]. These ten areas include communication; community use; functional academics; home living; health and safety; leisure; self-care; self-direction; sociability; work. The ABAS checklist can be completed by a parent in 15 minutes and has age-based norms and scaled scores and a general adaptive composite score with good validity and reliability [23].

*Conners' Parent Rating Scale-Revised: Short Version* (CPRS-R:S) is a standardized multiple-item parent-response questionnaire that provides age-based T-scores for oppositional behavior, inattentiveness, hyperactivity, and cognitive problems [24].

*Kaufman Brief Intelligence Test* (K-BIT), designed for people ages 4–99 years, is a standardized individually administered instrument for assessment of both verbal and nonverbal intelligence, yielding vocabulary, matrices, and intelligence quotient (IQ) composite scores. The K-BIT can be administered in 30 minutes, has age-based norms and scaled scores, and has good validity and reliability [25].

*Kaufman Test of Educational Achievement* (K-TEA), designed for youth ages 6–18 years, is a standardized individually administered assessment that measures educational achievement in reading, mathematics, and spelling. The K-TEA can be administered in 30 minutes and has age-based norms and scaled scores, with good validity and reliability [26].

*General Information Questionnaire* (GIQ) is a parent-response nonstandardized dichotomous questionnaire developed for this study to provide information regarding SES of the family and child and past medical and educational history of the child.

*Hollingshead Socioeconomic Status* is a standardized scale that measures SES attributed to caregiver education and occupation and was calculated based on the information collected on the GIQ.

### 2.4. Analysis

Quantitative data included standard scores for verbal IQ, nonverbal IQ, full-scale IQ composite, and adaptive skills scores (K-BIT and ABAS); T-scores for attention, hyperactivity, oppositional behavior and cognitive skills (CPRS-R:S); SES scores; GIQ. Independent samples *t*-tests were used to measure performance differences between HED and control groups for score discrepancy between subscales of adaptive behavior and IQ scores, between cognitive (K-BIT) and educational achievement (K-TEA) scores, and, on attention, hyperactivity, oppositional behavior, and cognitive skills on CPRS-R:S.

Pearson product moment correlation was used to test correlation between full-scale IQ on K-BIT and adaptive score on ABAS. Paired *t*-tests were used to test any significant differences between these two measures. Multivariate analysis of covariance (MANCOVA) was used to test differences between the two measures while controlling for gender, age, and groups. Frequencies were compared to determine HED subjects versus controls on the number of children with statistically significant differences (>20) between educational standard scores on K-TEA and full-scale IQ on K-BIT, the number of children with K-BIT composite standard score lower than 70, and the number of children with CPRS-R:S T-score above 70 on oppositional, cognitive, hyperactivity, and ADHD index subscales. Statistical analysis was performed using SPSS-Version 13. Statistical significance was set *P* < 0.05.

## 3. Results

### 3.1. Demographics

Our sample included 45 children with HED (age range 4–19 years, *x* = 9.75 years, SD = 4.02). Among these children, 35 were boys and 10 were girls, 42 self-identified as Caucasian and 3 as non-Caucasian. Parents of HED subjects had relatively high maximum-achieved education levels, with 62.5% of mothers and 50% of fathers having earned at least college degrees.

Our control group included 59 children (age range 4–14 years, *x* = 9.79 years, SD = 2.36). Among these children, 41 were boys and 18 were girls, all of whom identified as Caucasian. In this group, 62.9% of the mothers and 70.7% of the fathers had earned at least graduate degrees. Fathers' education level was higher in the control group than in the HED group (*P* = 0.02).

The two groups were comparable for gender, age, and Hollingshead SES. Characteristics of HED subjects and controls are summarized in Table 1. 

Chi-square and Fisher's exact tests were conducted to test between-group GIQ differences in the use of special resource support including reading, math, language therapy, speech therapy, and occupational therapy and to test between-group differences for previous diagnoses of learning disability, of mental disability (i.e., intelligence and adaptive functioning standard scores 2 or more standard deviations below the mean), and of ADHD. On Fisher's exact test, math resources showed a statistically significant difference between the two groups; five children in the HED group received resources for math versus none in the control group (*P* = 0.011). Further, on Fisher's exact test, there was a statistically significant difference for previous ADHD diagnosis, with 5 in the HED group versus 1 in the control group (*P* = 0.041). No other between-group differences were identified (Table 2).

### 3.2. Cognitive Assessment

Among subjects with HED, the distribution of K-BIT scores showed relatively high cognitive skills. None of the HED subjects or controls scored in the ranges of intellectual disability (IQ below 70) or borderline intelligence (IQ ranging 71–80). Results on K-BIT composite IQ scores ranging from above average to upper extreme were achieved by 52.6% of the subjects with HED and 55.6% of the controls. The youngest child (4 years old) in the HED group scored 51 on the nonverbal cognitive subscale and 116 on verbal cognitive subscale. Due to the significant discrepancy between this child's verbal and nonverbal cognitive scores, the full-scale IQ of 83 (below average) should be considered an underestimate of actual intelligence. Distribution of K-BIT composite IQ standard scores is shown in Table 3.

In the HED group, K-BIT IQ Composite score was in the range of low average (SS 80–89) in four children (9.5%), average (90–109) in 16 children (38.1%), above average (110–119) in 13 children (28.9%), well above average (120–129) in 7 children (15.6%), and upper extreme (>130) in two children (4.8%). In the control group, K-BIT IQ Composite score was in the range of low average in one child (1.8%), average in 25 children (46.7%), above average in 18 children (30.0%), well above average in 8 children (13.3%), and upper extreme in two children (3.3%).

**Figure 1:**
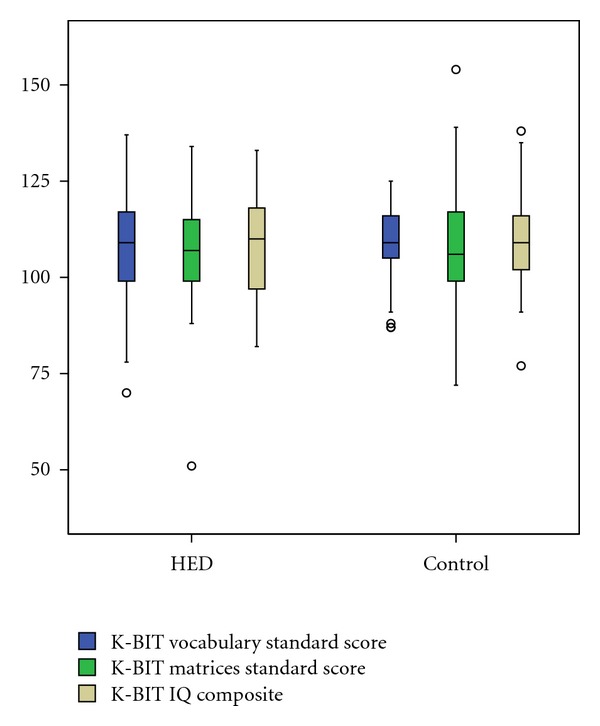
K-BIT standard scores for verbal (vocabulary), nonverbal (matrices), and full-scale (composite) intelligence quotient (IQ) among children with HED and control group.

There was no significant between-group difference for K-BIT mean verbal or nonverbal cognitive standard scores. Mean cognitive scores and standard deviations for the sample and controls are shown in Table 3 and Figure 1.

### 3.3. Educational Achievement Profile

For the purpose of this study, we defined learning disability as a 20-point or greater difference between verbal IQ (as measured by K-BIT vocabulary) and nonverbal IQ (as measured by K-BIT matrices), or as a 20-point or greater difference between full-scale IQ (as measured by K-BIT) and any individual achievement score including reading, math, and/or spelling (as measured by K-TEA). In the HED group, there were 10 (23.8%) children with significant difference (≥20) in K-BIT matrices less K-BIT vocabulary. In the control group, there were 14 children (25.5%) with significant difference (≥20) in K-BIT matrices less K-BIT vocabulary. There was no statistically significant between-group difference for children with significant matrices-less-vocabulary differences.

**Figure 2:**
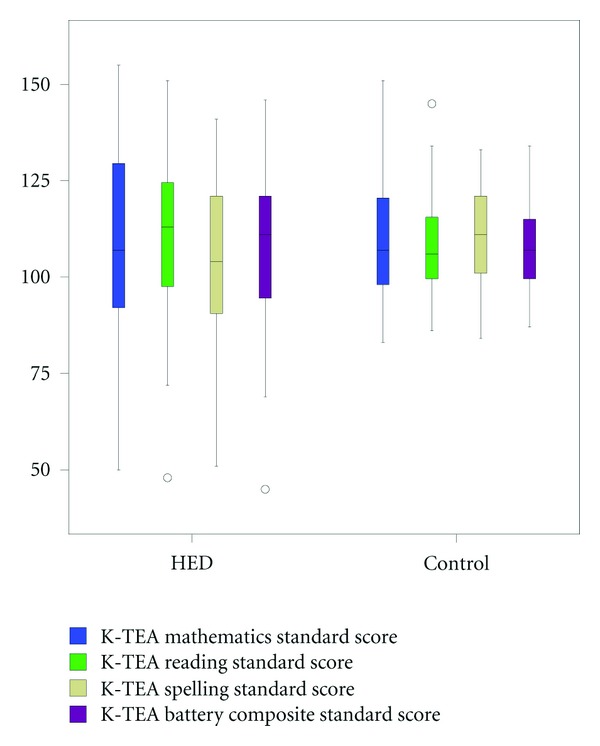
Comparison of K-TEA standard scores for children with HED and control group.

Mean achievement scores for the HED sample and controls are shown in Table 4 and Figure 2. There was no significant between-group difference for K-TEA reading scores or for K-TEA mathematics scores.

Children with HED had significantly lower K-TEA scores on spelling (*P* < 0.05) compared to scores achieved by controls. There was no significant difference between full-scale IQ on K-BIT when compared to educational achievement performance scores on reading, math, or spelling K-TEA subscales. Educational achievement performance among children with HED was consistent with their performance on this standardized measure of intelligence.

Parent education level of both HED subjects and controls correlated positively with performance on K-TEA math, K-BIT vocabulary, and K-BIT composite IQ (alpha = 0.05), and with performance on K-TEA reading and K-TEA battery composite (alpha = 0.01).

### 3.4. Adaptive Functioning

For the purpose of this study, mental disability was defined as significant impairment in intelligence and adaptive functioning with standard scores greater than two standard deviations below the mean. As shown before, in the HED group, there were no children who achieved a K-BIT full-scale IQ lower than 70.

We also calculated the difference between standard scores on K-BIT-IQ and ABAS general adaptive composite (GAC) by subtracting ABAS-GAC score from K-BIT-IQ score. A paired sample correlation and a paired *t*-test were conducted for both HED and control groups. On multivariate analysis of covariance (MANCOVA), when gender, age, and group were controlled, no statistically significant difference was found between K-BIT-IQ and ABAS-GAC scores. 

### 3.5. Attention Deficit Hyperactivity Disorder

#### 3.5.1. Comparison of Mean T-Scores on CPRS-R:S Subscales between HED and Control Groups

The four subscales of the CPRS-R:S were used to compare the two groups: opposition; cognition; hyperactivity; ADHD index. There was no statistically significant difference between the two groups in the T-scores on oppositional and cognitive subscales. There was a marginally significant difference (equal variances were not assumed by Levene's test) between the mean scores on hyperactivity (mean of HED = 54.59, SD of HED = 15.042, mean of control = 49.58, SD of control 8.071, *P* = 0.063) that approached statistical difference, and there was a significant difference in inattention scores assessed by ADHD index (mean of HED = 55.15, SD of HED = 11.753, mean of control = 49.33, SD of control = 8.406, *P* < 0.01) (Table 5).

#### 3.5.2. Number of Children with a T-Score above 70 on CPRS-R:S on Oppositional, Cognitive, Hyperactivity, and ADHD Index Subscales

Using a minimum cutoff T-score above 70 as significantly elevated on CPRS-R:S, five children with HED (12.8%) were rated as significant on the oppositional subscale, three (7.6%) on the cognitive subscale, seven (17.9%) on the hyperactivity subscale, and five (12.8%) on the ADHD index. In the control group, six children (10.5%) were rated as significant on the oppositional subscale, one (1.8%) on the cognitive subscale, one (1.8%) on the hyperactivity subscale, and three (5.2%) on the ADHD index. There is a sevenfold statistically significant difference in the other subscales, based on parent report (Table 6).

## 4. Discussion

Our results suggest that children with HED perform comparably to unaffected peers matched for age, gender, and SES on standardized measures of cognition and educational achievement, and have similar adaptive functioning but may be at increased risk for ADHD. Contrary to reports indicating as much as 50% prevalence of intellectual disability among people with HED, none of our participants had intellectual disability or borderline IQ, instead achieving relatively high scores on measures of cognition. Our study is the largest to date measuring these indices in children with HED and provides strong evidence challenging the notion that people with HED are at increased risk for cognitive impairment and/or learning disability.

Contributing factors that may falsely influence impressions of intellectual or academic abilities in children with HED include those common to many children who have chronic health conditions that can disrupt continuity in school attendance. In addition, children with HED have differences in physical appearance that may place them at increased risk for social-emotional challenges. Ecological theories of social perception indicate that people judge intelligence and character attributes based on facial qualities deemed attractive [27]. Positive attributes like friendliness and popularity are presumed based on pleasing dentofacial appearance [11], while negative attributes, such as less employability, intelligence, or trustworthiness, bias opinion unfavorably toward people with facial malformations [28]. As is true for other professionals, physicians are also prone to underestimate cognitive ability based on dysmorphic physical appearance [8].

The wide variability among past reports in the estimates of intellectual disability in people with HED (range 10–50%) may reflect significant methodological limitations, including isolated case reports of syndromes associated with HED or small sample sizes [9, 10, 17–19]. Case reports of college graduates with HED who have above average intelligence support our findings of above average intelligence in some people affected with HED [8]. Ours is the first reported study that has assessed cognitive function in children with HED using a standardized psychometric instrument with a relatively large sample size and frequency-matched control subjects.

Intellectual disability may be characterized along multiple cognitive domains, but legal definitions refer to performance on standardized instruments more than 2 standard deviations below the mean in both cognitive and adaptive functioning. We assessed adaptive skills indirectly by parent report, finding these reported functional levels in children with HED commensurate with their scores achieved in direct cognitive testing and without significant difference in either cognitive or adaptive profiles when compared with unaffected peers. Small-scale studies examining adaptive functioning in children and adolescents with HED have had mixed results. Similar to our findings, Tanner [21] found normal adaptive function according to parental report on ABAS scale in children with HED. Hummel and Guddak [29] found a range in adaptive function of children and adolescents with HED and identified multiple influences including the affected subject's own perceptions, quality of family support, and peer perception, as well as effects of disease state.

In assessing for learning disability, we adhered to limited objective criteria, which variably include as a primary criterion that scores on standardized measures of educational achievement in mathematics, reading, and/or written expression be substantially below (i.e., more than 2 standard deviations) that expected for age [22], relative to cognitive potential, as measured by IQ testing. In our study, this distinction required a 20-point or greater difference between scores on the K-BIT composite IQ and any one or more scores on the K-TEA scores (reading, mathematics, and/or spelling). There were no significant discrepancies between K-TEA reading score or math score when compared to K-BIT cognitive scores in either HED or control groups. The difference was slightly higher but not statistically significant in HED children. Children with HED had similar achievement scores when compared with controls except for weakness in spelling (*P* < 0.05). Children with HED showed a relative weakness in spelling scores when compared with this group's scores in reading and math. As there were no children in either group with a significant difference between composite IQ scores on K-BIT when compared to scores in K-TEA reading, mathematics, or spelling, none of our participants met the primary 20-point or greater difference criterion for learning disability. Results should be interpreted cautiously due to the small number of children who received resources in mathematics and to our narrow definition for learning disability applied in this study design.

We also considered the potential influence of ADHD in children with HED, as ADHD represents the most common neurobehavioral disorder of childhood (estimated prevalence is 5% of school-aged children) [30], and its presence may therefore contribute to perceptions of cognitive impairment and interfere with educational achievement. ADHD is underdiagnosed, with the National Institutes of Health reporting up to 50% of children with ADHD undiagnosed and untreated [31]. Children with ADHD are at substantially increased risk for poorer academic performance resulting from comorbid learning disabilities, cognitive impairment [32], and other neurodevelopmental disabilities, as well as from psychosocial and functional variables due directly and indirectly to inattention, impulsive behavior, distractibility, and poor concentration. In addition, children with ADHD are at increased risk for cognitive impairment [32]. In our study, we used the CPRS-R:S to screen for the presence of ADHD. The Conners' scales provide a standardized and well-accepted index for the assessment of ADHD, although the scales must be used in a clinical setting to support, rather than establish, a diagnosis of ADHD. Responses from parents of HED children were higher (i.e., more suggestive) in hyperactivity and inattentiveness (ADHD Index) subscales than were responses from parents of control subjects. There was a marginally significant difference between the mean scores on hyperactivity (*P* = 0.063) and a significant difference in inattentiveness (*P* < 0.01). Conversely, parents of children with HED reported less oppositional behavior than did parents of control subjects.

### 4.1. Limitations

Data from our study were acquired in nonclinical settings. Since ADHD is a diagnosis of exclusion, meaning that anxiety, mood disorder, psychosocial stress, sensory impairment, and other plausible explanations for the observed diagnostic features have been comprehensively considered and ruled out as fully explaining the behaviors, we were unable to provide a definitive diagnosis of ADHD for any study participant. In addition, lack of Conners' Teacher Rating Scales regarding HED children further limited our interpretable available data.

Another limitation of this study is the relatively high SES of our subjects. We do not know if this sample represents children with HED from all socioeconomic strata, as we tested only children who participated at the national family conference on ectodermal dysplasia. Children representing lower SES may be less likely to participate at the annual conference. Ascertainment bias is possible in that parents of children with HED who have true cognitive and learning deficits may not volunteer to participate in the study. Finally, diagnosis was not confirmed by genetic analysis, so that some subjects may have been genetically distinct from those with common X-linked recessive HED.

### 4.2. Future Research

Validation of ADHD profiles in children with HED will require more comprehensive assessments, including information from teachers or other observers and broader consideration of cultural influences. Exploration for endophenotypes and genetic susceptibilities may distinguish variations in risk for cognitive and behavioral impairment among HED-affected children.

### 4.3. Implications

Parents, teachers, and primary health care providers should be advised that HED is unlikely to be associated with intellectual disability or learning disability but may be associated with an increased risk for ADHD. Untreated ADHD may have a deleterious impact on learning and behavioral performance. Anticipation of possible bias in the community, at school, and elsewhere regarding limited intellectual potential due to facial or dental dysmorphic features should be recognized. Appropriate steps toward building skills in personal advocacy and promoting general awareness of accurate information about HED may be useful, although this consideration has not been formally or systematically assessed in HED.

### 4.4. Conclusions

This is the first reported study formally examining intelligence and educational achievement performance among children with HED. Our findings do not support reports of increased risk for intellectual impairment or learning disability among children with HED. Cognitive and educational achievement performance and adaptive functioning using standardized measures are similar in children with HED and unaffected controls matched by age, gender, and SES. However, parents of children with HED report increased inattentiveness and hyperactivity.

## Figures and Tables

**Table 1:** Demographic characteristics of children with HED and control group.

	HED (*n* = 45)	Controls (*n* = 59)	*P* value
Male gender (%)	78	70	NS
Mean age ± (years)	9.75 ± 4	9.79 ± 2.36	NS
Age range (years)	4–19	6–14	
Mothers with college and/or graduate degree (%)	62.5	62.9	NS
Fathers with college and/or graduate education (%)	50	70.7	0.02
Mean Hollingshead socioeconomic score	48.6	51.1	NS
Race			
(i) Caucasian (%)	93.4	100	NS
(ii) African American (%)	6.6	0	4.712

NS: not statistically significant.

**Table 2:** Previous diagnosis of ADHD and/or learning disability, and educational resources for children with HED and control group.

	HED (*n* = 45)	Controls (*n* = 59)	*P* value (Fisher's exact test)
Learning disability	4	2	NS
ADHD	5	1	0.041 (4.682)
Reading resources	5	3	NS
Math resources	5	0	0.011 (7.316)
Language therapy	3	2	NS
Speech therapy	6	3	NS
Occupational therapy	2	1	NS

NS: not statistically significant.

**Table 3:** Distribution of cognitive scores on K-BIT in children with HED and control group.

Range of composite IQ standard scores	HED (*n* = 45)(%)	Controls (*n* = 59)(%)	*P* value
Well below average (70–79)	0.0	1.8	NS
Below average (80–89)	9.5	0.0	NS
Average (90–109)	38.1	46.7	NS
Above average (110–119)	28.9	30.0	NS
Well above average (120–129)	15.6	13.3	NS
Upper extreme (130–160)	4.8	3.3	NS
Missing data	3.3	4.9	NS

NS: not statistically significant.

**Table 4:** Comparative distribution of standard scores on IQ (K-BIT) and educational achievement (K-TEA) in children with HED and control group.

	HED (*n* = 45) mean (standard deviation)	Controls (*n* = 59) mean (standard deviation)	*P* value
K-BIT			
(i) IQ composite	107.49 (21.142)	109.70 (11.883)	NS
(ii) Vocabulary	107.64 (15.284)	109.26 (9.338)	NS
(iii) Matrices	106.88 (15.129)	108.02 (16.219)	NS
K-TEA			
(i) Battery composite	107.49 (21.142)	107.53 (10.612)	NS
(ii) Reading	111.66 (20.643)	107.98 (11.710)	NS
(iii) Mathematics	109.03 (24.616)	109.44 (15.364)	NS
(iv) Spelling	104.49 (19.993)	110.65 (12.108)	0.05

NS: not statistically significant.

**Table 5:** Mean T-scores on CPRS-R:S in children with HED and control group.

	HED (*n* = 39)	Controls (*n* = 57)	*P* value
Oppositional	49.72	50.89	NS
Cognitive	50.82	49.02	NS
Hyperactivity	55.154 ± 15.042	49.58	0.063
Inattentiveness	55.59 ± 11.753	49.43 ± 8.45	0.01

NS: not statistically significant.

**Table 6:** Percentage of abnormal T-Scores on CPRS-R:S in children with HED and control group.

	HED (*n* = 39) %	Controls (*n* = 57) %	*P* value
Oppositional	12.8	10.5	NS
Cognitive	7.6	1.8	NS
Hyperactivity	17.9	1.8	0.01
Inattentiveness	12.8	5.2	NS

NS: not statistically significant.

## Abbreviations

AAIDD: American Association on Intellectual and Developmental Disabilities
ABAS: Adaptive Behavior Assessment Scale
ABAS-GAC: ABAS-General Adaptive Composite
ADHD: Attention deficit hyperactivity disorder
CPRS-R:S: Conners' Parent Rating Scale-Revised: Short Version
DSM-IV-TR: Diagnostic and Statistical Manual of Mental Disorders, 4th edition, Text Revision
GIQ: General Information Questionnaire
HED: Hypohidrotic ectodermal dysplasia
IQ: Intelligence quotient
K-BIT: Kaufman Brief Intelligence Test
K-TEA: Kaufman Test of Educational Achievement
NFED: National Foundation for Ectodermal Dysplasias
SES: Socioeconomic status.
